# Development of a bipolar disorder biobank: differential phenotyping for subsequent biomarker analyses

**DOI:** 10.1186/s40345-015-0030-4

**Published:** 2015-06-24

**Authors:** Mark A Frye, Susan L McElroy, Manuel Fuentes, Bruce Sutor, Kathryn M Schak, Christine W Galardy, Brian A Palmer, Miguel L Prieto, Simon Kung, Christopher L Sola, Euijung Ryu, Marin Veldic, Jennifer Geske, Alfredo Cuellar-Barboza, Lisa R Seymour, Nicole Mori, Scott Crowe, Teresa A Rummans, Joanna M Biernacka

**Affiliations:** Department of Psychiatry and Psychology, Mayo Clinic, 200 First St SW, Rochester, MN 55905 USA; Division of Biomedical Statistics and Informatics, Mayo Clinic, Rochester, MN USA; Lindner Center of HOPE, Mason, OH USA; Department of Psychiatry, University of Cincinnati College of Medicine, Cincinnati, OH USA; Department of Psychiatry, Universite Desarrollo and Clinca Allemana, Santiago, Chile; Department of Psychiatry, Universidad de los Andes, Santiago, Chile; Department of Psychiatry, Autonomous University of Nuevo Leon, Monterrey, Mexico; Department of Psychiatry, University of Minnesota, Minneapolis, MN USA; Department of Psychiatry and Psychology, Mayo Clinic, Jacksonville, FL USA

**Keywords:** Biobank, Bipolar disorder, Phenotype

## Abstract

**Background:**

We aimed to establish a bipolar disorder biobank to serve as a resource for clinical and biomarker studies of disease risk and treatment response. Here, we describe the aims, design, infrastructure, and research uses of the biobank, along with demographics and clinical features of the first participants enrolled.

**Methods:**

Patients were recruited for the Mayo Clinic Bipolar Biobank beginning in July 2009. The Structured Clinical Interview for DSM-IV was used to confirm bipolar diagnosis. The Bipolar Biobank Clinical Questionnaire and Participant Questionnaire were designed to collect detailed demographic and clinical data, including clinical course of illness measures that would delineate differential phenotypes for subsequent analyses. Blood specimens were obtained from participants, and various aliquots were stored for future research.

**Results:**

As of September 2014, 1363 participants have been enrolled in the bipolar biobank. Among these first participants, 69.0 % had a diagnosis of bipolar disorder type I. The group was 60.2 % women and predominantly white (90.6 %), with a mean (SD) age of 42.6 (14.9) years. Clinical phenotypes of the group included history of psychosis (42.3 %), suicide attempt (32.5 %), addiction to alcohol (39.1 %), addiction to nicotine (39.8 %), obesity (42.9 %), antidepressant-induced mania (31.7 %), tardive dyskinesia (3.2 %), and history of drug-related serious rash (5.7 %).

**Conclusions:**

Quantifying phenotypic patterns of illness beyond bipolar subtype can provide more detailed clinical disease characteristics for biomarker research, including genomic-risk studies. Future research can harness clinically useful biomarkers using state-of-the-art research technology to help stage disease burden and better individualize treatment selection for patients with bipolar disorder.

## Background

Bipolar disorder is a medical illness characterized by recurrent episodes of mania or hypomania and major depression (Kraepelin 1921; Goodwin and Jamison 2007). Historically, the lifetime prevalence has been reported as ≈1 %, and the standard treatment, lithium, has been adequate. However, in the past 20 years, increasing evidence has suggested higher prevalence rates (up to 5 %, including subsyndromal diagnostic criteria) and has shown subgroups with differential response to alternative mood-stabilizing treatments (Kessler et al. 2005; Frye 2011).

Bipolar disorder is highly heritable, and additive genetic effects can account for up to 85 % of the variance in risk (Bienvenu et al. 2011). However, not all disease-risk genes have been fully identified. Recent genome-wide association studies have identified several relatively diverse risk genes, including *Ankyrin-G* or *ANK3* (encoding ankyrin 3) (Ferreira et al. 2008), *NCAN* (encoding neurocan) (Cichon et al. 2011), *DGKH* (encoding diacylglycerol kinase) (Baum et al. 2008), *CACNA1C* (voltage-dependent calcium channel) (Ferreira et al. 2008; Sklar et al. 2008), *ODZ4* (human homologue of *Drosophila* gene) (Psychiatric GWAS Consortium Bipolar Disorder Working Group 2011), *TRANK1* (tetratricopeptide repeat and ankyrin repeat containing 1) (Mühleisen et al. 2014), and *ADCY2* (adenylate cyclase 2) (Mühleisen et al. 2014). Further investigation has also focused on enriched samples (e.g., pediatric cohorts with early-onset disease and positive family history), which has identified candidate genes such as the clock gene *RORB* (RAR-related orphan receptor beta) (McGrath et al. 2009). Further research using large collections of patients with precisely defined phenotypes and high-quality specimens, which are noted barriers to effective clinical translation (Olson et al. 2013), can potentially improve clinical management of bipolar disorder by developing genetic testing for disease risk and pharmacogenomic testing to guide treatment.

Mayo Clinic, in collaboration with the Lindner Center of HOPE and the University of Minnesota, established a bipolar disorder biobank. The biobank uses state-of-the-art research technology to allow for both clinical and biomarker studies of bipolar disease risk and treatment response. This article describes (1) the aims, design, and infrastructure of the bipolar biobank; (2) demographics and clinical features of the first 1363 participants enrolled; and (3) examples of research uses of the biobank and similar resources.

## Methods

### Administrative oversight

The Mayo Clinic Bipolar Biobank was initiated in 2009; key collaborating sites included the Lindner Center of HOPE/University of Cincinnati and the University of Minnesota. The co-principal investigators (M.A.F. and J.M.B.) provide administrative oversight, along with the biobank’s executive committee, the Mayo Clinic Biospecimen Trust Oversight Group, and the Center for Individualized Medicine (Olson et al. 2013).

### Recruitment and informed consent

Enrollment sites, each with site-specific institutional review board approval, included Mayo Clinic, Rochester, Minnesota; Lindner Center of HOPE/University of Cincinnati College of Medicine, Cincinnati, Ohio; and the University of Minnesota, Minneapolis, Minnesota. Potential participants were identified through various methods, including routine clinical appointments, in-patients admitted in mood disorder units, and recruitment advertising. Participants were required to be between 18 and 80 years old and be able to speak English, provide inform consent, and have DSM-IV-TR diagnostic confirmation of type I or II bipolar disorder or schizoaffective bipolar disorder (American Psychiatric Association 2000). Patients with active psychosis or active suicidal ideation were not invited to participate in the biobank. The written informed consent process was followed by a comprehension test questionnaire to ensure key points of study participation were understood (i.e., longevity of DNA sample, deidentified samples in studies within and outside of primary institution, potential conflict of interest, Frye et al. 2015b).

### Demographic and clinical data collection

Tools used to evaluate the participants included the Structured Clinical Interview for DSM-IV (SCID) modules A, D, and overview, a Bipolar Biobank clinical questionnaire designed to assess clinical variables such as course of the illness, past treatments and response rates, and psychiatric or medical comorbid conditions, and a Bipolar Biobank participant questionnaire for demographic characteristics (e.g., marital status, race and ethnicity, education, and occupational functioning level), family history, and current substance use.

Course of illness measures were collected and focused on the phenotype domains of psychosis, suicidality, mood instability, comorbid anxiety, and comorbid substance abuse. A participant was positive for psychosis if he or she had a lifetime history of hallucinations or delusions, specified in mania, depression, or both or outside of episodes. Suicidality was designated as positive if the patient had one or more attempts requiring medical intervention. Mood instability was a composite sum (range, 0–5) of the lifetime presence (yes = 1, no = 0) of five features: mixed episodes, rapid cycling, ultrarapid/ultradian cycling, cycle acceleration over time, and increased severity of episodes over time. Comorbid anxiety was a similar composite sum (range, 0–6) of the lifetime presence of six features: posttraumatic stress disorder, generalized anxiety disorder, social anxiety disorder, obsessive-compulsive disorder, phobia, and panic disorder. Comorbid substance abuse was the sum (range, 0–3) of the lifetime prevalence of three features: alcohol abuse or dependence, drug abuse or dependence, and nicotine dependence.

Obesity was defined as a body mass index (BMI) of 30 kg/m^2^ or more and extreme obesity as a BMI of 40 kg/m^2^ or more. The Cumulative Illness Rating Scale (CIRS) (Linn et al. 1968) was used to assess current or past medical illness burden. Severity was designated as follows: 0, none: no impairment from or problem with that system; 1, mild: current mild or past significant problem; 2, moderate: impairment interferes with normal activity; 3, severe: severe problems and/or disabling impairment and/or hard-to-control chronic problems; 4, extremely severe: life threatening. The CIRS has been validated as a measure of medical burden in major depression (Papakostas et al. 2003) and bipolar disorder (Kemp et al. 2009, 2014), with high medical illness burden defined as a total CIRS score of 4 or greater. For this study, the CIRS was modified by removing the “psychiatric illness” item, resulting in 13 organ system–oriented questions. All clinical phenotype is in clinical database separate from the biospecimens.

### Biospecimen collection

Venipuncture was performed using standard techniques. A total of 45 mL of blood was collected from each participant. Blood was drawn into two 10-mL EDTA tubes, one 10-mL no-additive serum tube, one 10-mL sodium heparin tube, and one 4.5-mL sodium citrate tube. All tubes were labeled with a study subject identifier, collection date, and time of draw. After collection, samples were electronically accessioned at the Biospecimens Accessioning Processing Laboratory at the Mayo Clinic Advanced Genomics Technology Center. Samples underwent subsequent fractionation, DNA extraction, analysis, and storage. All disease related biobanks at Mayo Clinic and the Community Biobank (Olson et al. 2013) have standardized all procedures for specimen collection, DNA extraction, serum/plasma processing, and storage to enable case vs control analyses that are matched for specimen quality control.

### Statistical analysis

Participants’ demographic characteristics obtained from the participant questionnaire and clinical variables obtained from the SCID and the clinical questionnaire were described with standard summary statistics. Statistical summaries were prepared using SAS software version 9.3 (SAS Institute, Inc).

## Results

Among the first 1363 participants enrolled in the bipolar biobank through September 2014, the racial makeup was predominantly white (90.6 %), 60.2 % were women, and the mean (SD) age was 42.6 (14.9) years (Table 1). Although a majority of participants (84.6 %) had attended at least some college, more than half (55.7 %) were not currently employed.

**Table 1:** Demographics of participants in the bipolar disorder biobank (*n* = 1363)

Characteristic	Value^a^
Women	820 (60.2)
Age at enrollment, y	42.6 (14.9)
Race	(*n* = 1336)
White	1211 (90.6)
Black	35 (2.6)
Multiracial	55 (4.1)
Other	35 (2.6)
Education level	(*n* = 1296)
Less than high school	34 (2.6)
High school graduate or GED	166 (12.8)
Some college or higher	1096 (84.6)
Employment	(*n* = 1274)
Working	564 (44.3)
Not currently working for pay	710 (55.7)
Marital status	(*n* = 1298)
Married/cohabitating	625 (48.2)
Separated/divorced	240 (18.5)
Widowed	29 (2.2)
Single	404 (31.1)

*GED* general educational development tests

^a^Values are no. of patients (%) or mean (SD)

The specific diagnoses of the participants were bipolar disorder I in 69.0 %, bipolar disorder II in 29.2 %, and schizoaffective bipolar disorder in 1.8 % (Table 2). Current psychiatric comorbidity was high, most notably with generalized anxiety disorder (41.4 %), nicotine dependence (26.6 %), panic disorder (20.1 %), social anxiety disorder (18.9 %), attention deficit hyperactivity disorder (17 %), posttraumatic stress disorder (16.1 %), alcohol abuse/dependence (14.1 %), and bulimia/anorexia/binge eating disorder (2.7 %). Lifetime comorbid conditions (Table 2) included generalized anxiety disorder (50.7 %), nicotine dependence (39.8 %), alcohol abuse/dependence (39.1 %), panic disorder (31.3 %), attention deficit hyperactivity disorder (27.8 %), posttraumatic stress disorder (26.4 %), social anxiety disorder (24.6 %), and bulimia/anorexia/binge eating disorder (10.4 %).

**Table 2:** Clinical characteristics of participants in the bipolar disorder biobank (*n* = 1363)

Characteristic	Value^a^
SCID diagnosis	
Bipolar I	941 (69.0)
Bipolar II	398 (29.2)
Schizoaffective bipolar	24 (1.8)
Current or recent depression	679 (49.8)
History of psychosis	563 (42.3)
	(*n* = 1330)
Suicide attempt	436 (32.5)
	(*n* = 1340)
Mood instability	
Mean value	1.5 (1.4)
	(*n* = 1333)
Mixed mania	134 (14.0)
	(*n* = 954)
Rapid cycling	699 (52.0)
	(*n* = 1343)
Ultrarapid	379 (28.3)
	(*n* = 1338)
Cycle acceleration	353 (26.5)
	(*n* = 1330)
Increased severity	446 (33.4)
	(*n* = 1336)
Anxiety disorder	
Mean value	1.5 (1.4)
	(*n* = 1333)
Posttraumatic stress disorder	345 (26.4)
	(*n* = 1307)
Generalized anxiety disorder	664 (50.7)
	(*n* = 1309)
Social anxiety disorder	319 (24.6)
	(*n* = 1297)
Obsessive-compulsive disorder	192 (14.7)
	(*n* = 1309)
Phobia	131 (10.1)
	(*n* = 1301)
Panic disorder	409 (31.3)
	(*n* = 1306)
Addiction, lifetime	
Mean value	1.0 (1.0)
	(*n* = 1342)
Alcohol abuse/dependence	514 (39.1)
	(*n* = 1316)
Nicotine dependence	522 (39.8)
	(*n* = 1313)
History of drug/substance	343 (27.0)
	(*n* = 1271)
BMI	(*n* = 1257)
Mean value, kg/m^2^	29.91 (6.95)
Overweight	397 (31.6)
Obese	421 (33.5)
Morbid obesity	118 (9.4)
First-degree relative suicide	125 (11.7)
	(*n* = 1067)
Antidepressant-induced mania	247 (31.7)
	(*n* = 779)
Tardive dyskinesia	37 (3.2)
	(*n* = 1167)
Drug-related rash	65 (5.7)
	(*n* = 1147)
Serious rash	9 (3.7)
	(*n* = 246)

*BMI* body mass index, *SCID* Structured Interview for DSM-IV

^a^Values are no. of patients (%) or mean (SD)

At the time of enrollment in the biobank, almost half the patients had a current or recent depressive episode (49.8 %) (Table 2). Additional clinical phenotypes included history of psychosis (42.3 %), history of one or more suicide attempts requiring medical attention (32.5 %), composite score of mood instability (1.5 (1.4)), comorbid anxiety composite score (1.5 (1.4)), drug addiction composite score (1.0 (1.0)), family history of completed suicide (11.7 %), and several adverse drug-related events (antidepressant-induced mania (31.7 %), tardive dyskinesia (3.2 %), history of drug-related rash (5.7 %), and serious rash (3.7 %)).

**Fig. 1:**
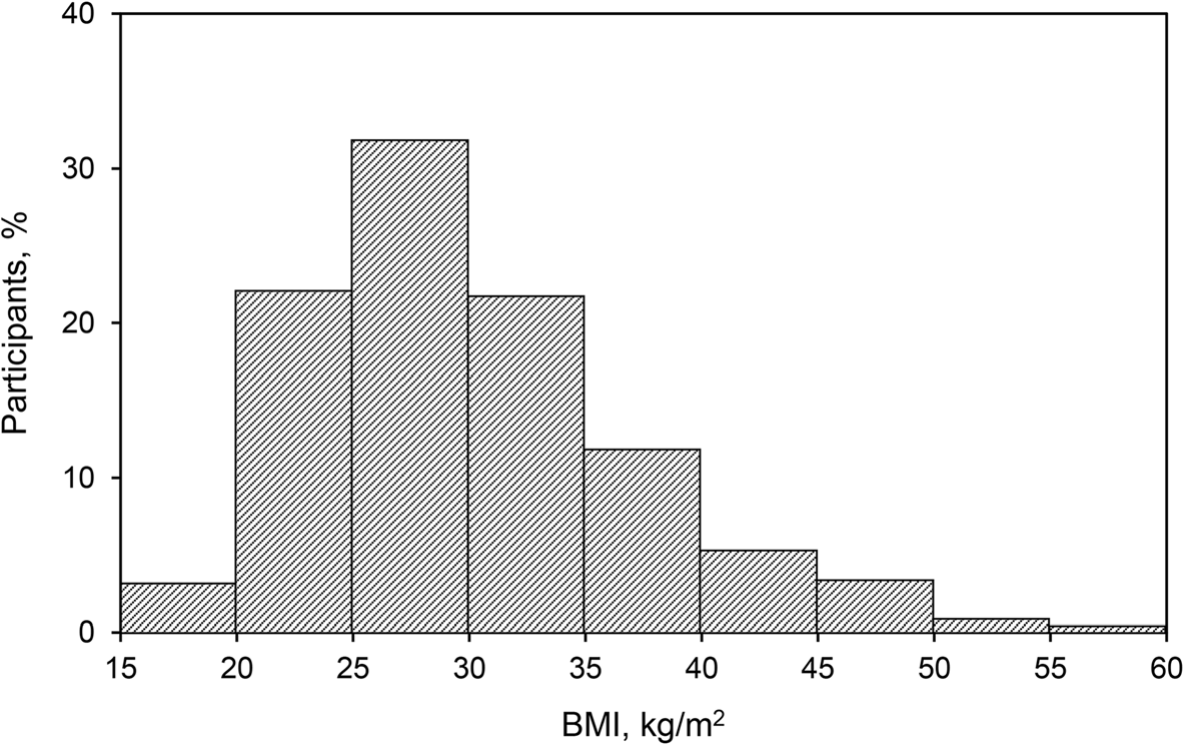
Body mass index of the participants (*n* = 1257)

Medical comorbid conditions were substantial. The total mean (SD) CIRS was 4.1 (3.6) with subsection mean scores as follows: cardiac, 1.2 (0.6); hypertension, 1.4 (0.7); vascular, 1.2 (0.5); respiratory, 1.4 (0.7); eyes, ears, nose, throat, 1.4 (0.7); lower gastrointestinal, 1.3 (0.6); upper gastrointestinal, 1.4 (0.7); hepatic, 1.1 (0.3); renal, 1.1 (0.4); other genitourinary, 1.3 (0.6); musculoskeletal, 1.5 (0.8); neurologic, 1.6 (0.8); and endocrine-metabolic, 1.5 (0.8). A high medical burden (total score ≥4) was present in 47.7 % of participants. A total of 539 participants (42.9 % of 1257 with data available) met the criteria for obesity, with a BMI of 30 kg/m^2^ or higher (Fig. 1).

## Discussion

This initial evaluation of the demographic and clinical characteristics of patients with bipolar disorder enrolled in the Mayo Clinic Bipolar Disorder Biobank suggests a group with substantial psychiatric and medical comorbidity. The demographics and comorbid conditions of the study sample are representative of the general population of patients with bipolar disorder and, thus, potential research findings based on this sample would be generalizable. For example, the mean age and depressive-predominant disease are very similar to those reported in regulatory treatment trials for bipolar depression (McElroy et al. 2010). The percentages of comorbid anxiety, addiction, and obesity resemble other published demographics in longitudinal studies from the Stanley Foundation Bipolar Network (Leverich et al. 2001; Suppes et al. 2001) and the Systematic Treatment Enhancement Program for Bipolar Disorder (Kogan et al. 2004). The prevalence rates of high medical burden resemble other studies in bipolar disorder (Kemp et al. 2014), with rates of obesity higher than in the general population (Ogden et al. 2014).

An important strength of the biobank is the detailed phenotype obtained for all participants. The detailed quantified phenotype—that is, the composite measures of mood instability, comorbid anxiety, and multiple drug addiction, beyond the SCID-confirmed bipolar disorder subtype—represents novel composite quantifications and may provide more detailed clinical disease characteristics that can be used in future biomarker genomic studies. Our group has already identified risk genes not previously identified from datasets in the public domain by narrowing the phenotype from bipolar disorder to a specific comorbid condition, which in essence decreases phenotypic (and therefore genetic) heterogeneity (Winham et al. 2014). We have since replicated these findings using samples from the Mayo Clinic Biobank (Cuellar-Barboza et al.). Future studies that focus not on a DSM5 bipolar disorder diagnosis but on a more detailed phenotype such as bipolar disorder with obesity may uncover risk genes not previously identified.

Narrowly defined phenotypes may also provide greater power in pharmacogenomic studies of treatment response or adverse events. An example is The Consortium for Lithium Genetics, the goal of which is to facilitate high-quality, well-powered genomic analyses of lithium treatment response (Schulze et al. 2010) with a narrow standardized phenotype of prophylactic lithium response quantified using the Alda Scale (Grof et al. 2002). As another example, early work from our group focused on developing a narrowly defined phenotype of antidepressant-induced mania. Whereas most prior pharmacogenetic studies of antidepressant-induced mania focused on association with the s allele of the promoter region length polymorphism (5-HTTLPR) in the serotonin transporter gene *SLC6A4* (Biernacka et al. 2012), our group identified a unique *SLC6A4* haplotype composed of the 5-HTTLPR, SNP rs25531, and intron 2 VNTR that was associated with a decreased risk of this adverse drug event (Frye et al. 2015a). Such pharmacogenomics studies have the potential to transform clinical practice. Such transformation has already been seen with the mood-stabilizing agent carbamazepine, which underwent a US Food and Drug Administration boxed warning revision when an association between HLA-B*1502 and the risk of serious dermatologic adverse effects (Stevens-Johnson syndrome, toxic epidermal necrolysis) was identified in persons of Han Chinese descent (Ferrell and McLeod 2008). The HLA-B*1502 allele was not identified in other races or ethnicities, but recent data identified similar HLA antigens, HLA-B*1511 and HLA-A3101, in Stevens-Johnson syndrome and severe cutaneous reactions in a Korean population (Kim et al. 2011).

Because of the demographics in the recruitment areas, the biobank sample currently has little racial and ethnic variation, being 90 % white. Although this has advantages in terms of reducing heterogeneity in biomarker studies, new discoveries will need to be confirmed in other specific or more diverse populations. It will be important for future studies to analyze replication cohorts, particularly in study samples of more diverse ancestry. Our biobank is broadening study sites with a particular focus on persons with ancestry other than European in other parts of the USA, as well as in Mexico and Chile.

The biobank data are limited by some retrospective aspects of illness quantification and lack of current state-dependent measures of illness severity. Both of these are being addressed with a prospective biomarker clinical trial embedded within the biobank, with rating scales for depression and mania.

This phenotypically rich resource will continue to enroll new subjects and encourage discovery of genomic and other biomarkers using state-of-the-art research technology to help stage disease burden and better individualize treatment selection. The breadth of data and biospecimen availability (anticipate expansion to RNA and other bio specimen such as CSF) from biobank participants will provide the opportunity for numerous and diverse research collaborations with high potential for clinical translation. In a time of unprecedented growth in the scope of medical research, which necessitates increased efficiency, data and samples can no longer be collected for the purpose of performing a single focused study. Current technologies allow for the measurement of various blood biomarkers (including DNA genetic variants, metabolites, and proteins); this produces high-dimensional data that allow for a broad range of hypothesis-generating and hypothesis-testing studies. Large scale collaborative research will need to employ easy to implement information technology to ensure the success of these research initiatives (Demiroglu et al., 2012).

## Conclusions

Given the complexities in diagnosing bipolar disorder, particularly in adolescents and young adults, as well as high rates of morbidity and mortality, there is great clinical need for improved diagnostics. Genomic medicine may provide the tools to assist clinicians in making a diagnosis and increase the likelihood of earlier successful treatment interventions. Future studies would benefit from including a unipolar cohort with longitudinal follow-up to better understand genomic disease-risk prediction. Furthermore, biomarkers of treatment response may enable clinicians to target the right drug to each patient, thus, minimizing unsuccessful treatment trials. Identifying biomarkers can also help with risk stratification for adverse events related to various treatments and enhance drug development, for an increased and individualized bipolar pharmacopoeia.

## Abbreviations

BMI: body mass index
CIRS: Cumulative Illness Rating Scale
SCID: Structured Clinical Interview for DSM-IV
